# Accident injuries of children and adolescents in Germany. Results of the cross-sectional KiGGS Wave 2 study and trends

**DOI:** 10.17886/RKI-GBE-2018-086.2

**Published:** 2018-09-19

**Authors:** Anke-Christine Saß, Ronny Kuhnert, Johanna Gutsche

**Affiliations:** Robert Koch Institute, Berlin, Department of Epidemiology and Health Monitoring

**Keywords:** ACCIDENT, INJURY, CHILDREN AND ADOLESCENTS, HEALTH MONITORING, KIGGS

## Abstract

For children and adolescents, accidents represent an important health risk. Despite decreasing mortality rates, accidental (unintended) injuries remain the most common cause of death for children over the age of one in Germany. Accident injuries can cause considerable and lasting damage on health and development. The possible major implications as well as the potential to prevent accident injuries underline the importance of accident prevention. The German Health Interview and Examination Survey for Children and Adolescents (KiGGS) collects data on unintentional child injuries at regular intervals. Results of the second follow-up survey (KiGGS Wave 2, 2014-2017) show that during the past twelve months 16.5% of children and adolescents aged between 1 and 17 received medical treatment following an accident. Boys suffer injuries from accidents significantly more often than girls (18.6% vs. 14.3%). While the prevalences for older children and adolescents tend to be higher, age generally has little impact on accident rates. Compared to the two previous waves of KiGGS, the prevalences of accident injuries have remained stable.

## Introduction

For children and adolescents, accident injuries represent a considerable health risk. Despite decreasing mortality rates, accident injuries remain the leading cause of death for children aged one or older in Germany and Europe [1]. In 2015, 281 children and adolescents in Germany died in accidents. 182 of these accident victims were aged under 15, 99 were adolescents aged between 15 and 17 (Code V01-X59 of the International Statistical Classification of Diseases and Related Health Problems, 10th revision, ICD-10) [2]. Injuries, a substantial proportion of which are caused by accidents, are among the most common reasons for children and adolescents seeking medical attention in hospital. In 2016, 199,300 children up to the age of 15 and 48,000 adolescents aged 15 to 17 received treatment in hospital due to injury (ICD-10 S00-T98, excluding surgical complications T80-T88). Depending on the age group, this makes injuries the second most important or even the leading cause for hospital admissions at childhood and adolescent age (for children over the age of one) [3].

High treatment costs as well as temporary or permanent functional limitations, pain and loss of quality of life explain the major public health relevance of accident prevention. According to the World Health Organization, most unintentional and intentional injuries could be avoided [4]. However, preventing accident injuries requires detailed information on accidents as well as on their determining factors [5].

**KiGGS Wave 2**

Second follow-up to the German Health Interview and Examination Survey for Children and Adolescents

**Data owner:** Robert Koch Institute

**Aim:** Providing reliable information on health status, health-related behaviour, living conditions, protective and risk factors, and health care among children, adolescents and young adults living in Germany, with the possibility of trend and longitudinal analyses

**Study design:** Combined cross-sectional and cohort study

**Cross-sectional study in KiGGS Wave 2**

**Age range:** 0-17 years

**Population:** Children and adolescents with permanent residence in Germany

**Sampling:** Samples from official residency registries - randomly selected children and adolescents from the 167 cities and municipalities covered by the KiGGS baseline study

**Sample size:** 15,023 participants

**KiGGS cohort study in KiGGS Wave 2**

**Age range:** 10-31 years

**Sampling:** Re-invitation of everyone who took part in the KiGGS baseline study and who was willing to participate in a follow-up

**Sample size:** 10,853 participants

**KiGGS survey waves**

► KiGGS baseline study (2003-2006), examination and interview survey
► KiGGS Wave 1 (2009-2012), interview survey
► KiGGS Wave 2 (2014-2017), examination and interview survey

More information is available at www.kiggs-studie.de/english

Germany has a heterogeneous data basis for accident injuries. Data is systematically collected only for specific fields of non-fatal accidents (school and road traffic accidents), but not for other fields like accidents at home or during leisure time [6]. Up-to-date epidemiologic data is important to identify and evaluate the most pressing fields of action. In addition to official statistics and the routine data provided by social insurance carriers, population surveys contribute to the description and analysis of accidents [6]. Within the framework of recurrent questionnaire-based surveys, the German Health Interview and Examination Survey for Children and Adolescents (KiGGS) collects information on non-fatal accident injuries [7-9].

## Indicator

KiGGS forms part of health monitoring at the Robert Koch Institute and includes regular cross-sectional surveys on children and adolescents aged 0 to 17 that are representative of the German population (KiGGS cross-sectional component). After having carried out the baseline study as an interview and examination survey between 2003 and 2006, and KiGGS Wave 1 as an interview-based survey between 2009 and 2012, KiGGS Wave 2 was implemented between 2014 and 2017 as a combined interview and examination survey.

A detailed description of the methodology can be found in New data for action. Data collection for KiGGS Wave 2 has been completed in issue S3/2017 as well as KiGGS Wave 2 cross-sectional study – participant acquisition, response rates and representativeness in issue 1/2018 of the Journal of Health Monitoring [10, 11].

KiGGS wave 2 surveyed accidents prevalence based on data provided by parents or legal guardians from a questionnaire answered in writing. The relevant question was, ‘Has your child been treated by a doctor in the last 12 months due to an injury (e.g. following an accident, poisoning or violence)?’ They could answer either ‘Yes’ or ‘No’. To differentiate between unintentional and intentional injuries, respondents answering ‘Yes’ were then asked: ‘Was/were this/these injury/injuries or poisoning(s)…’ ‘… unintentional, as an accident?’ or ‘… result of violence during a physical confrontation?’ For children older than three years of age there was also the category of deliberate self-harm. For this article, the analysis only considers unintentional injuries.

The findings presented here are based on the data relating to 14,141 children and adolescents (7,082 girls, 7,059 boys) aged 1 to 17 with valid data on injuries for which they received medical treatment. Infants were excluded to ensure comparability with previous waves of KiGGS. This age group was excluded from the KiGGS baseline study and KiGGS Wave 1 due to the small number of cases that prohibit a more in-depth analysis. For the most recent KiGGS Wave 2, too, the number of reported accident injuries for children aged up to one year is small (n=13).

The results are presented as prevalences (frequencies) and are stratified by gender, age and socioeconomic status. Family socioeconomic status was measured through an index based on the information parents provided on educational background, occupational status and income situation (equivalised disposable income) [12].

The calculations were carried out using a weighting factor that corrects deviations within the sample from the population structure with regard to age in years, gender, federal state, German citizenship and the parents’ levels of education (Microcensus 2013 [13]). P-values to demonstrate linear trends across the KiGGS survey waves were calculated using univariate logistical regression and was based moreover on age-standardised prevalences (as at 31 December 2015).

**Info box 1: Injuries in official statistics**

In Germany, official statistics record information on injuries according to the 10th revision of the International Statistical Classification of Diseases and Related Health Problems (ICD-10).

Chapter XIX (S00 – T98) Injury, poisoning and certain consequences from external causes

► The affected body region and type of injury is coded, no distinction is made between intentional and unintentional injuries (accidents)

Chapter XX (V01–Y98) External causes of morbidity and mortality

► Possibility of differentiating between intentional and unintentional injuries (accidents), is only used to code causes of death (cause of death statistics)

This article considers prevalences with 95% confidence intervals (95% CI). A statistically significant difference between groups is assumed to have been demonstrated with p-values of less than 0.05.

## Results and discussion

Over the past twelve months 16.5% of children and adolescents aged between 1 and 17 received medical treatment for an accidental injury (Table 1). This is about one in six children. The proportion is higher for boys (18.6%) compared to girls (14.3%). This difference is statistically significant.

Accident frequency tends to increase with age. Overall, however, the differences between age groups are small. A statistically significant difference is only evident between the 3 to 6 age group and the group of children and adolescents above eleven years of age (Table 1). Injury frequency varies by socio-economic status of families. Among adolescents from the low status group, an accident injury tended to be less frequently reported than among adolescents from the other two status groups. The findings are significant in boys across both status comparisons, while significant among girls when comparing low and high status group.

A comparison of data from the most recent wave of KiGGS and the previous KiGGS baseline study (2003-2006) and KiGGS Wave 1 (2009-2012) shows that accident prevalences and figures for age groups and genders have remained fairly constant (data not shown). The finding that boys more often are affected by accident injuries compared to girls is evident in all three waves of KiGGS, which is reflected in the official causes of death, hospital and road accident statistics [1, 5, 7, 8].

In the previous waves of KiGGS, no correlation between the frequency of accidental injuries (total) and family socioeconomic status was found [5, 7, 8]. Only road traffic accidents surveyed in the KiGGS baseline study show a marginal increase in injury rate for girls and boys from families with low socioeconomic status [7]. Regional studies from Germany highlight this correlation for road traffic accidents and scalding (see overview [7]). Further analyses are necessary to explore the influence of the social situation on accident occurrence.

Fortunately, the number of children and adolescents who suffer fatal accident injuries has been decreasing for years in Germany [2], current KiGGS data nonetheless indicate that non-fatal accidents continue to represent a high risk for the health of children and adolescents. The wide implications of accident injuries and the potential to prevent them underline the importance of accident prevention. Identifying particularly vulnerable population groups and accident hotspots requires comprehensive information on how injuries occur. The KiGGS study asks additional questions about the incident of injury such as the accident site and products involved in causing the injury for example. The corresponding data available from earlier KiGGS waves reflect the lives and living environments of children and adolescents, and facilitate a differentiated view of accident locations and products involved [7-9]. KiGGS Wave 2 will allow to update this information.

## Key statements

- 16.5% of children and adolescents aged between 1 and 17 years received medical treatment for accident injuries during the last twelve months.
- Boys suffer injuries from accidents significantly more often than girls.
- Compared to the two previous waves of KiGGS, accident injury prevalences have remained stable.

## Figures and Tables

**Table 1:** Prevalence of medically treated accident injuries during the past twelve months according to gender, age and socioeconomic status (n=7,082 girls, n=7,059 boys) Source: KiGGS Wave 2 (2014-2017)

KiGGS Wave 2		
	%	(95% CI)
**Girls (total)**	**14.3**	**(13.2-15.4)**
**Age group**		
1-2 Years	12.5	(9.0-17.1)
3-6 Years	11.3	(9.5-13.5)
7-10 Years	13.0	(11.2-15.0)
11-13 Years	17.8	(15.2-20.7)
14-17 Years	16.4	(14.3-18.7)
**Socioeconomic status**		
Low	11.3	(8.6-14.7)
Medium	14.7	(13.4-16.0)
High	16.0	(14.0-18.1)
**Boys**	**18.6**	**(17.4-19.8)**
**Age group**		
1-2 Years	17.3	(13.2-22.4)
3-6 Years	14.9	(12.7-17.3)
7-10 Years	17.4	(15.4-19.7)
11-13 Years	21.8	(19.0-24.8)
14-17 Years	21.3	(18.9-24.1)
**Socioeconomic status**		
Low	15.0	(12.4-18.2)
Medium	19.3	(17.8-20.9)
High	20.5	(18.2-23.1)
**Total (girls and boys)**	**16.5**	**(15.7-17.3)**

CI = confidence interval
