# Corrigendum: Accident injuries of children and adolescents in Germany. Results of the cross-sectional KiGGS Wave 2 study and trends

**DOI:** 10.25646/6802

**Published:** 2019-09-13

**Authors:** Anke-Christine Saß, Ronny Kuhnert, Johanna Gutsche

Saß AC, Kuhnert R, Gutsche J (2018)

Journal of Health Monitoring 3(3): 50-55.

DOI 10.17886/RKI-GBE-2018-086

This fact sheet presents the 12-month prevalence of unintentional (accidental) injuries. An earlier version of this article failed to separate accidental from intentional injuries (violence or self-injury) due to an error in variable filtering. As a result, the number of study participants included in the analyses and the reported prevalences were slightly higher than expected.

The corrected version of the article is available at DOI 10.17886/RKI-GBE-2018-086.2

